# Lipidome of Endemic Fish *Comephorus dybowskii* (Scorpaeniformes, Comephoridae) in Lake Baikal Plays an Essential Role in Maintaining Organism Homeostasis Due to Biomembrane Modifications and Metabolic Consistency

**DOI:** 10.3390/membranes16080269

**Published:** 2026-08-13

**Authors:** Viktor P. Voronin, Ekaterina D. Voronina, Anna A. Etingova, Sergey I. Didorenko, Nina N. Nemova, Svetlana A. Murzina

**Affiliations:** 1 Institute of Biology of the Karelian Research Centre of the Russian Academy of Sciences (IB KarRC RAS), 11 Pushkinskaya Street, 185910 Petrozavodsk, Russia; katya.tushina@gmail.com (E.D.V.); nnnemova@gmail.com (N.N.N.); murzina.svetlana@gmail.com (S.A.M.); 2 Federal State Budgetary Scientific Institution “Baikal Museum of the Siberian Branch of the Russian Academy of Sciences”, Irkutsk Region, Irkutsk District, Listvyanka Settlement, Akademicheskaya Street, 1, 664520 Listvyanka, Russia; maritui@mail.ru (A.A.E.); didorenkos@mail.ru (S.I.D.)

**Keywords:** lipid metabolism, fatty acids, membrane remodeling, little Baikal oilfish, *Comephorus dybowskii*, cold adaptations, endemic species, Lake Baikal

## Abstract

The lipid and fatty acid composition of the little Baikal oilfish (*Comephorus dybowskii* Korotneff, 1905) was studied to identify the features of its biochemical adaptation to the environmental conditions of Lake Baikal. Multivariate analysis of lipid classes and FA profiles revealed reproducible clustering patterns in both muscle tissue and whole-body samples, indicating the presence of distinct physiological states differing in membrane organization and lipid metabolism. Lipid-class variability was primarily associated with sphingomyelin, cholesterol esters, phosphatidylinositol, and lysophosphatidylcholine, suggesting the coordinated regulation of membrane structure. The fatty-acid profiles were mainly presented by variations in saturated, monounsaturated, and long-chain polyunsaturated fatty acids, with muscle tissues characterized by a relatively longer carbon chain (ACL = 19 vs. 18 I whole body) and unsaturation (UI = 1.98–2.76 vs. 1.64–2.53 in whole body). At the same time, comparative analysis demonstrated low correspondence between lipid and FA profiles, indicating partially independent adaptive mechanisms. The experimental transfer of fish from their natural habitat to controlled conditions resulted in the significant remodeling of both lipid and FA compositions, including increases in acylglycerols, membrane phospholipids, and monounsaturated fatty acids, together with a decline in long-chain n-3 polyunsaturated fatty acids. The results suggest that lipid classes and fatty acids represent complementary but functionally distinct levels of biochemical adaptation contributing to the maintenance of membrane organization and metabolic homeostasis in *C. dybowskii*, revealing a previously undescribed multi-level organization of lipid-related adaptive responses in this endemic deep-water fish.

## 1. Introduction

Lipids and fatty acids (FAs) are among the most labile and sensitive organic molecules, designed to maintain homeostasis inside the organism exposed to changes in the exterior environment [1,2,3,4]. We know, in particular, that the degree of unsaturation of FA radicals within membrane phospholipids (PLs) is directly dependent on the ambient temperature, salinity, and/or hydrostatic pressure [3,5,6,7]. The accumulation of low-density substances (e.g., waxes, cholesterol esters (CholEs), or triacylglycerols (TAGs)) in aquatic organisms promotes positive buoyancy [8], even in bladderless fish [9,10]. Some lipidic components (e.g., arachidonic acid (cis20:4(n-6)), mono- and diacylglycerols (MAGs and DAGs), etc.) are often regarded as secondary metabolites or as their metabolic precursors, which are involved in many of the organism’s responses and compensatory reactions [4,11,12].

**Figure 1:**
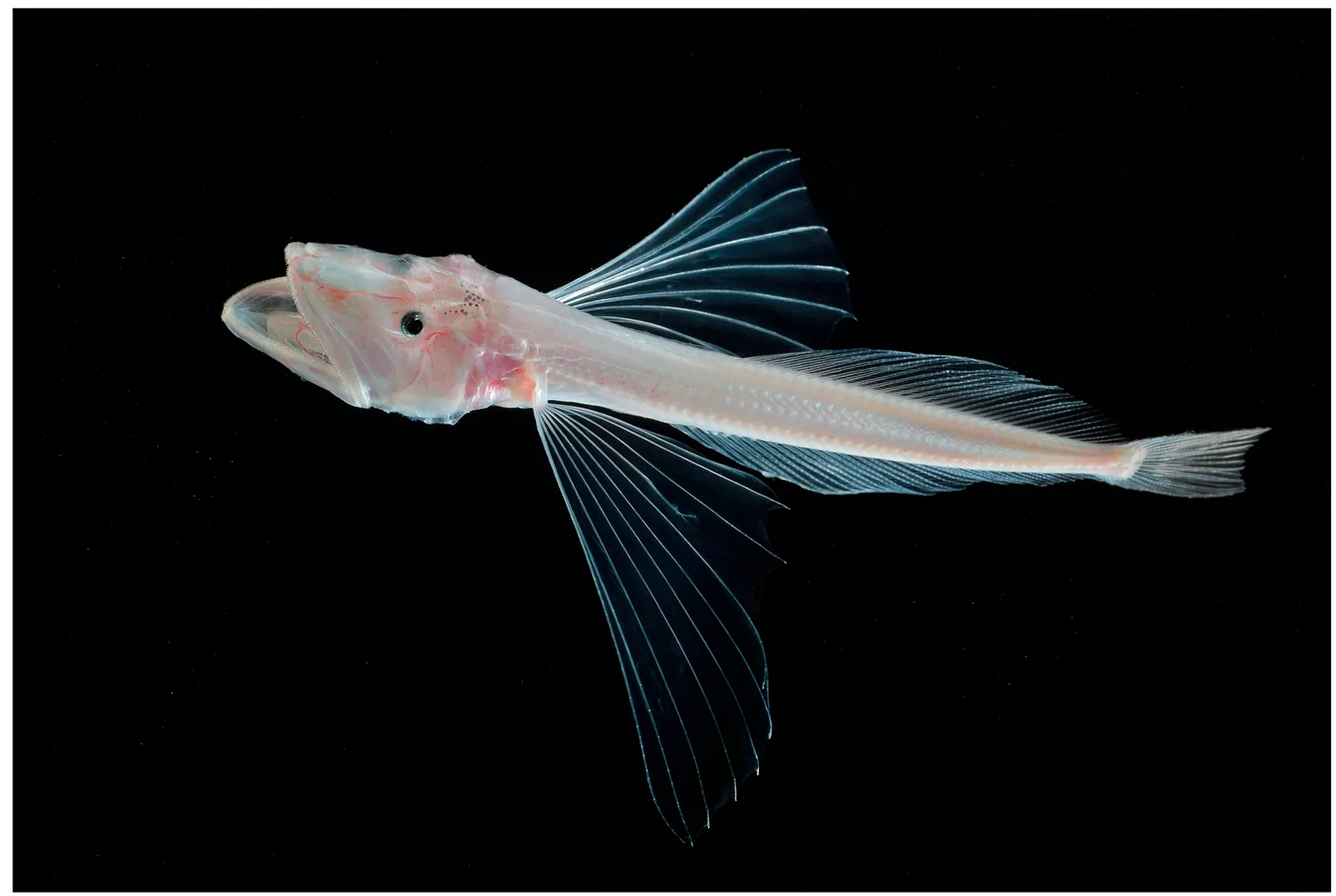
The adult *Comephorus dybowskii* Korotneff, 1904 is an endemic species of the pelagic zone of Lake Baikal. Photo by S.I. Didorenko.

Lake Baikal is a unique inland water body of tectonic genesis, with the environmental conditions varying widely from the water edge to the deepest regions [13]. The current diversity of Baikal’s fish fauna amounts to over 60 species and subspecies of 15 families, represented by demersal and pelagic forms [14]. One of them is the little Baikal oilfish, *Comephorus dybowskii* Korotneff, 1905 (Scorpaeniformes, Comephoridae) (Figure 1), which is one of the most abundant endemic species in the pelagic zone of Lake Baikal [15,16]. According to some reports, *C. dybowskii* abundance in the 1960s–70s was over 40 billion individuals, i.e., ≈50% of the biomass of Baikal’s entire fish community [16,17]. The abundant *C. dybowskii* is the main food item for many organisms living in this region, while their scattering through the water column (without massive aggregations) is a hindrance for commercial fisheries. Little Baikal oilfish are also food for valuable coregonids, such as the omul, and constitute up to a half of the diet of the endemic Baikal seals [18].

Even if the biology of *C. dybowskii* bears certain similarity with that of other sculpin species living in Baikal, it is a secondary pelagic ecological form, having evolved from a demersal to a pelagic lifestyle through morphophysiological adaptations—the specific body density and skeletal weight has decreased, the hydrodynamic streamlining of the body has improved, fin physiology has been modified to increase the gliding surface, and the body color has changed [18,19]. Owing to these metamorphoses, *C. dybowskii* managed to occupy nearly the entire range of depths, from 10 to 1637 m [18]. Although the swim bladder has not re-appeared in Baikal oilfish, the species is still capable of daily and seasonal migrations across the water column [18]. Vertical movements, in this case, are restrained by the ambient conditions, specifically the water temperature. Abiotic factors are known to have a strong impact on the life and vitality of *C. dybowskii* [18]—a water temperature of 4–5 °C is optimal for this species, while temperatures above +9 °C are lethal [13]. That said, the mechanisms through which *C. dybowskii* adapt to abiotic impacts and their depth-related variation have remained poorly studied, especially at the molecular (biochemical) level. While some previous lipid profiling studies are available for *C. dybowskii* (e.g., [18,20,21]), these studies have focused on both studies on the lipid profile and biochemical adaptation based on lipid accumulation and variability in *Comephorus* species. However, the adaptive significance of the lipid composition has remained largely unexplored. In particular, no studies have simultaneously examined lipid classes and FA profiles as complementary levels of biochemical adaptation, nor have they evaluated their relative organization using modern multivariate approaches. Furthermore, experimental evidence of the lipidome responses of *C. dybowskii* to abrupt environmental change is currently lacking. Baikal oilfish represents a particularly informative model for such analyses because it is an endemic pelagic species inhabiting a broad depth range and regularly exposed to pronounced gradients of temperature and hydrostatic pressure. The combination of ecological specialization, a high lipid content, and vertical migrations allow the study of this species for lipid-related mechanisms of environmental adaptation. The present study therefore combines lipid classes and FA profiling with multivariate statistical analyses and experimental manipulation to identify previously undescribed levels of biochemical adaptation in this endemic deep-water fish.

In view of this, the aim of the study was to investigate the biochemical mechanisms of the lipid-metabolism adaptation in *C. dybowskii* to the variable environmental conditions of Lake Baikal. Revealing these mechanisms in endemic fish species through the case study of the little Baikal oilfish will provide new insights into the adaptation mechanisms that are already known for more widespread fishes, including marine and oceanic species.

## 2. Materials and Methods

**Figure 2:**
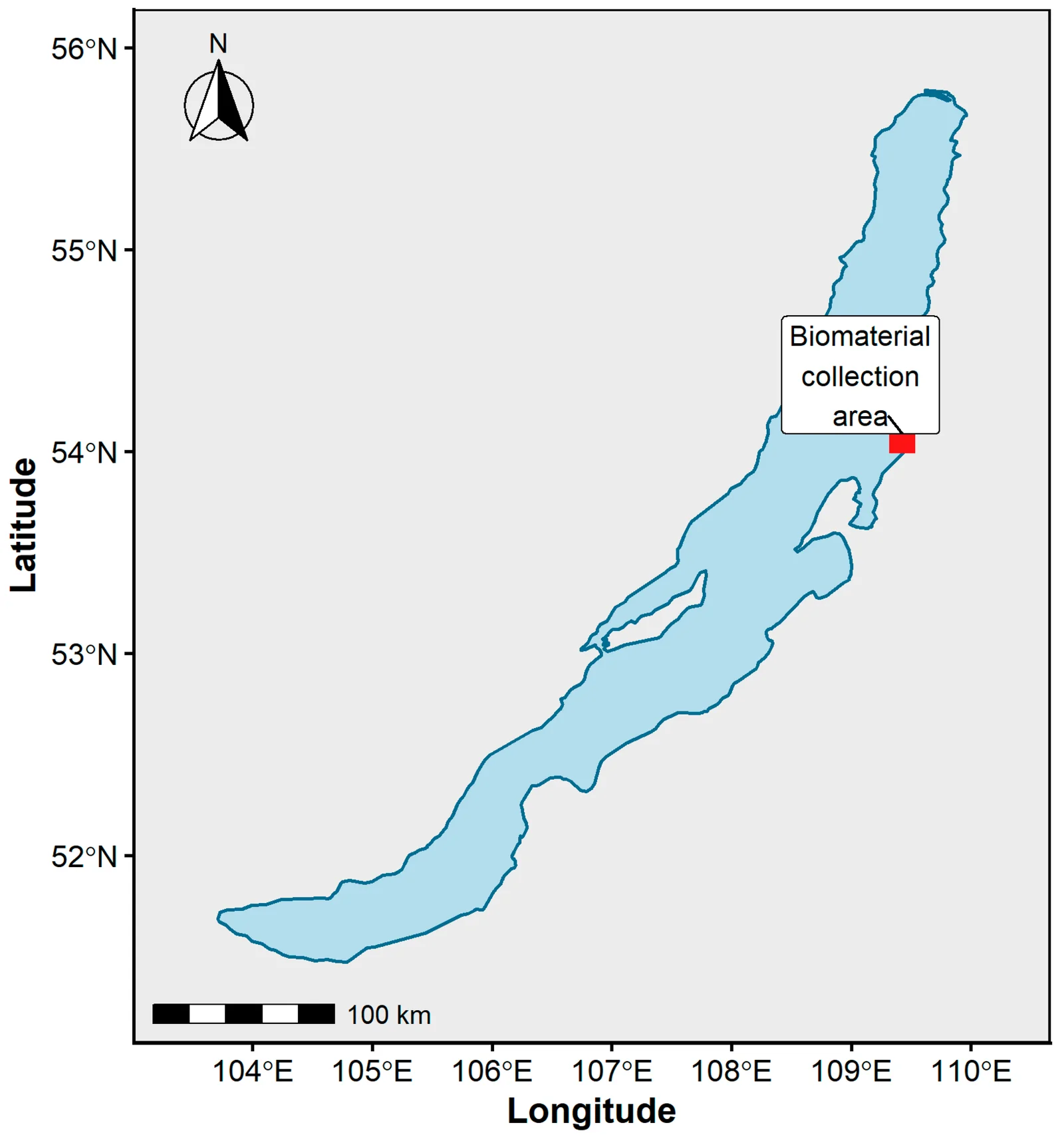
Schematic map of the collection region of the *Comephorus dybowskii* Korotneff, 1904 in Lake Baikal.

Biological samples of adult mature *C. dybowskii* were collected by trawling an area in the northern basin of Lake Baikal (Figure 2) in summer (July 2024) from aboard the “Professor A.A. Treskov” research vessel. The trawling gear was a demersal beam trawl of 100 cm width and 50 cm height. The *C. dybowskii* sampled for biochemical analysis weighed 1.24–3.53 g, with the body length between 7.1 and 9.3 cm. To reduce biological variability unrelated to environmental adaptation, only adult mature individuals were included in the study. All specimens were collected from the same region of Lake Baikal during a single sampling campaign (July 2024), thereby minimizing seasonal variation. Although potential differences in the feeding history, physiological condition, and reproductive status could not be fully controlled in nature (during the expedition), the relatively narrow range of body size and the simultaneous collection of individuals were expected to reduce their dependence on the observed lipid and fatty-acid patterns. The lipid analysis was performed using white muscle tissue biopsied from each individual (since fish of the genus *Comephorus* lack the lateral-line red muscle strip [20]), as well as the whole body. The fish were dissected directly after capture, and the specimens were fixed individually in liquid nitrogen and stored until arrival in the laboratory.

Additionally, six fish were placed, for 24 h, in controlled-environment tanks (5‰ salinity, 3–6 °C temperature) to monitor the organism’s compensatory adjustments to the new abiotic conditions. After 24 h, muscle tissue for lipid biochemical analysis was biopsied from these individuals as well. The prepared specimens were fixed in liquid nitrogen (−197 °C) until arrival in the laboratory. The subsequent biochemical studies were performed at the Laboratory of Ecological Biochemistry using equipment of the Karelian Research Centre’s Core Facility.

### 2.1. Lipid Extraction from Tissues

Total lipids (TLs) were extracted from homogenized tissues with a 2/1 (*v*/*v*) chloroform/methanol mixture using the Folch method [22]. The resulting TL extracts were separated into lipid classes via chromatography. The content of TLs was determined gravimetrically.

### 2.2. High-Performance Thin-Layer Chromatography (HPTLC)

Total PLs, MAGs, DAGs, TAGs, cholesterol and its esters (Chol and CholEs, respectively), non-esterified fatty acids (NEFAs), and waxes were isolated via HPTLC using a CAMAG HPTLC system (Switzerland). Total lipids were fractionated on HPTLC Silicagel 60 F_254_ Premium Purity glass plates (Merck, Darmstadt, Germany). Sample micro quantities (2 µL) were applied in narrow bands using the spray-on technique using a Linomat 5 semi-automatic sample applicator (CAMAG, Muttenz, Switzerland), and the applied analytes were then eluted in an ADC2 automatic chromatographic gradient development chamber (CAMAG, Switzerland). The mobile phase was the hexane/diethyl ether/acetic acid (32/8/0.8 *v*/*v*) organic solvent mixture [23]. The isolated lipid classes were visualized (stained) in an air-tight derivatizer (CAMAG, Switzerland) by spraying 2 mL of a copper sulfate (CuSO_4_) aqueous solution acidified with phosphoric acid (H_3_PO_4_) through the nozzle, followed by warming the plate to 160 °C for 15 min. The stained plates were scanned in the chamber of a TLC Scanner 4 densitometer (CAMAG, Switzerland) in adsorption mode at a 360 nm wavelength. The qualitative and quantitative parameters of the lipid components were determined taking into account the mobility values for the corresponding analytical standards (Sigma-Aldrich, Burlington, MA, USA).

### 2.3. High-Performance Liquid Chromatography (HPLC)

Some of the main PL classes—phosphatidylcholine (PC), phosphatidylethanolamine (PE), phosphatidylserine (PS), phosphatidylinositol (PI), lysophosphatidylcholine (LysoPC), and sphingomyelin (SM)—were isolated from the TL mix via HPLC using a “Stayer” liquid chromatographer (Aquilon, Podolsk, Russia). Total PLs were fractionated on a 250 × 4 mm Nucleosil 100-7 column (Elsiko, Moskow, Russia) with an acetonitrile/methanol/hexane/85% H_3_PO_4_ mixture (918/30/30/17.5 *v*/*v*) as the mobile phase [24], at a 1 mL/min flow rate. The isolated components were identified in adsorption mode at a 206 nm wavelength. The qualitative and quantitative parameters of individual PL classes were also determined taking into account the mobility values for the corresponding analytical standards (Sigma-Aldrich, USA).

### 2.4. Gas Chromatography (GC)

The FA profile of *C. dybowskii* was analyzed via GC using chromatographers fitted with single-quadrupole mass-selective (GC-MS) and flame ionization (GC-FID) detectors. Immediately preceding the analysis, the TL mix was subjected to acid methylation to produce fatty acid methyl esters (FAMEs). The FAME mix was separated via chromatography on an HP-88 60 m × 0.25 mm × 0.20 µm capillary column (Agilent Technologies, Santa Clara, CA, USA) with the following temperature program: t (initial) = 140 °C (hold for 5 min), ramp at 4 °C/min to 240 °C, t (final) = 240 °C (hold for 2 min). The mobile phase was helium in the GC-MS method and nitrogen for GC-FID. Individual FAs in the spectrum were identified with “Maestro α-MS” GC-MS (Interlab, Moscow, Russia) both in the full ion scanning mode (Scan-mode) in the 50 to 400 m/z range and by monitoring selected ions (SIM-mode) that characterize the FAs included in the analytical mixes Supelco 37, Bacterial Acid Methyl Ester (BAME) Mix, and PUFA No. 1 Marine source (Sigma-Aldrich, USA). The output was analyzed using “Maestro Analytic v. 1.025” software and the NIST’17 library. The GC-MS detection of FAs was followed by their quantification using “Chromatec Crystal 5000.2” GC-FID (Chromatec, Yoshkar-Ola, Russia) under identical chromatographic settings. The output was processed using “Chromatek Analytic v. 3.0.298.1” software. The quantification was based on plotting the linear regression for calibration, where the external standard was the Supelco 37 mix (Sigma-Aldrich, USA) with known concentrations. The response factor (*R_i_*) for quantifying the FAs not included in the Supelco 37 mix was calculated using the following formula:

$$R_{i}=\frac{M_{r}(n_{i}-1)}{M_{i}(n_{r}-1)}$$

where *M_r_* is the molar mass of the 16:0 FA internal standard, g/mol; *n_i_* is the number of carbon atoms in FA *i*; *M_i_* is the molar mass of FA *i*, g/mol; and *n_r_* is the number of carbon atoms in the 16:0 FA internal standard.

### 2.5. Statistical Treatment

Statistical treatment of the data employed multivariate techniques for compositional data analysis. Because the analyzed datasets were compositional and characterized by relatively small sample sizes, non-parametric and permutation-based statistical approaches were preferentially applied. Consequently, data normality was not considered a prerequisite for the main analyses. The initial values for the contents of lipid classes and FAs were first subjected to centered log-ratio (CLR) transform to adequately account for their compositional nature and eliminate the closure effect. The structure of variability in the lipid and FA composition was identified via principal component analysis (PCA). The analysis was run with CLR-transformed data, with a subsequent determination of the contributions of variables to the principal components and interpretation of the ordination axes. Clustering was done via hierarchical agglomeration using Ward’s method and the Euclidean distance in CLR space. The optimal number of clusters was determined using the silhouette index. The significance of differences between clusters was estimated via a non-parametric permutational multivariate analysis of variance (PERMANOVA) and analysis of similarities (ANOSIM). The relationship of lipid and FA profiles with morphometric characteristics and sampling depth was measured via PERMANOVA and coefficients of determination (R^2^). Comparisons between the spatial structure of lipid and FA profiles were drawn using Procrustes analysis and distance matrix correlations (Spearman’s test). Agreement between clustering partitions was evaluated using the Adjusted Rand Index (ARI) and Adjusted Mutual Information (AMI). Differences in the content of individual lipid classes between the ‘control’ and the ‘experiment’ were analyzed via the non-parametric test Wilcoxon–Mann–Whitney. The Benjamini–Hochberg procedure (FDR control) was used to adjust the *p*-value when doing multiple comparisons. The effect size was calculated as the *r* coefficient.

Each fish individual was treated as an independent biological replicate. Lipid and fatty-acid compositions were determined for each biological sample separately, and statistical analyses were performed using biological replicates. Technical measurements were used only for analytical quantification and were not treated as independent observations in statistical analyses. The lipid and fatty-acid profiles of muscle tissue were analyzed in 10 individuals, whereas whole-body analyses included 11 individuals. Experimental comparisons involved six fish transferred to controlled conditions and the corresponding control specimens collected from the natural habitat.

Data were processed and visualized in the R programing language in the RStudio (version 1.3.1056) development environment using specialized statistical analysis packages.

## 3. Results

The TL content in muscle tissues of *C. dybowskii* ranged from 0.77 to 4.82% dry weight (dw) across the 450–550 m depth range (Table 1). Muscle TLs were found to be composed of PLs accounting for 0.18–1.23% dw, storage lipids (TAGs+CholEs+waxes—0.46–4.13% dw), and Chol (0.10–0.90% dw) and minor lipid classes (MAGs: 0.01–0.04%; DAGs: 0.02–0.13%; NEFAs: 0.07–0.47% dw) (Table 1). In whole-body samples, the TL content varied among individuals from 2.28 to 14.75% dw, with 0.30–1.25% contributed by PLs, 1.56–10.13% by storage lipids (TAGs+CholE+waxes), 0.24–1.66% by Chol, and 0.18–1.71% by minor lipid classes (MAGs+DAGs+NEFAs) (Table 1).

**Figure 3:**
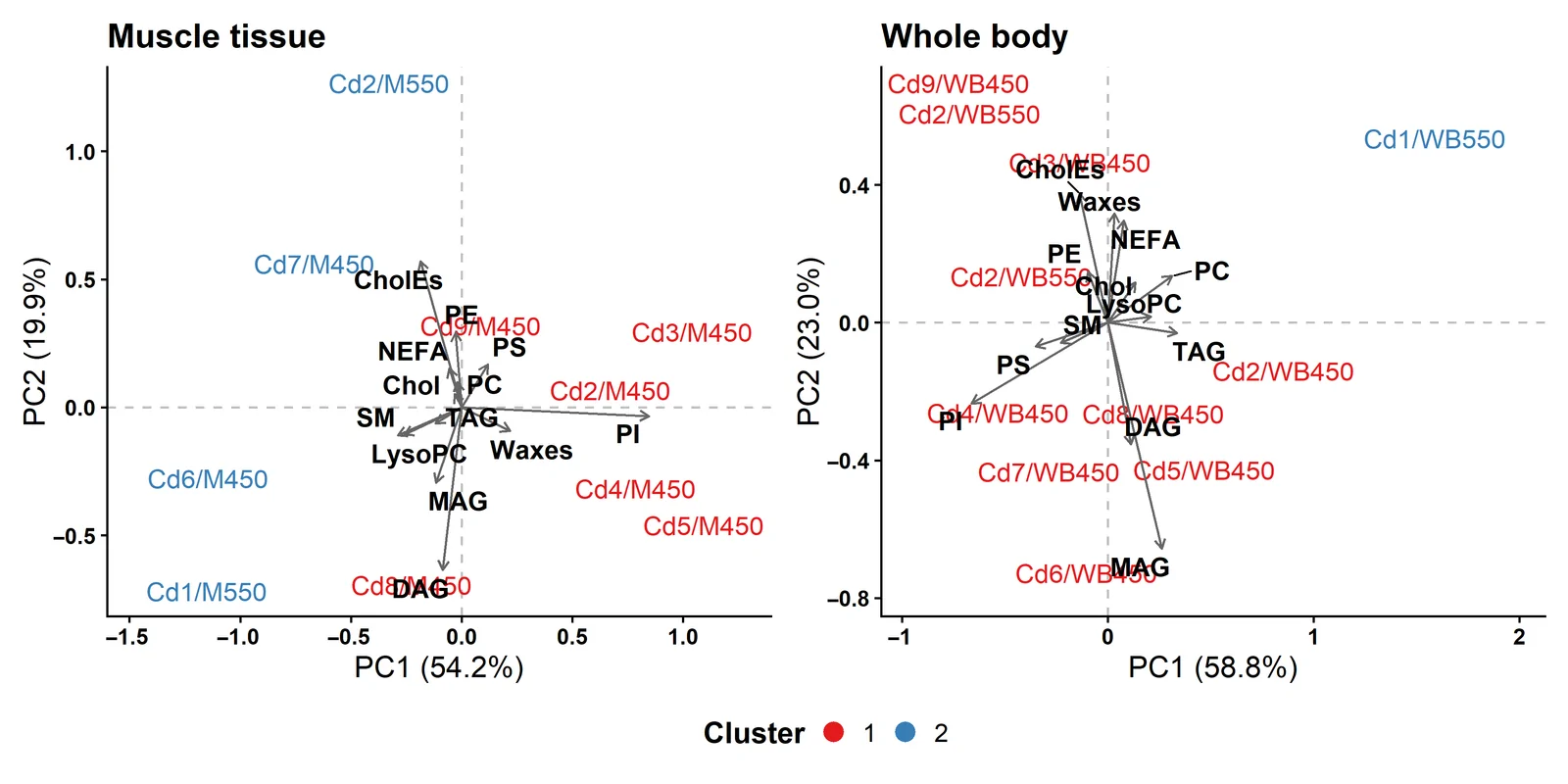
PCA ordination of *C. dybowskii* individuals based on the muscle tissue and whole-body lipid profiles in CLR space, with clustering based on Ward’s method. Note: TLs—total lipids, PLs—total phospholipids, TAGs—triacylglycerols, CholEs—cholesterol esters, waxes—wax esters, Chol—cholesterol, MAGs—monoacylglycerols, DAGs—diacylglycerols, NEFAs—non-esterified (free) fatty acids, PC—phosphatidylcholine, PE—phosphatidylethanolamine, PI—phosphatidylinositol, PS—phosphatidylserine, LysoPC—lysophosphatidylcholine, SM—sphingomyelin. Fish identity legend (Cd2/M450 as the example): Cd—*C. dybowskii*, 2—ID number of fish, M—muscles (WB—whole body), 450 (or 550)—capture depth (m).

The statistical treatment of data based on individual lipid classes (MAGs, DAGs, TAGs, Chol, CholEs, NEFAs, and waxes) and phospholipids (PC, PE, PS, PI, LPC, and SM) via PCA and hierarchical clustering (Ward’s method), preceded by CLR transform of compositional biochemical data (expressed as % dw), differentiated the lipid profiles of *C. dybowskii* individuals into two clusters for both muscle tissue and the whole body (Figure 3). In particular, the muscle lipid profile model explains 74.1% of the total data variance (PC1—54.2%, PC2—19.9%) with the two most optimal profile clusterings using Ward’s method (k = 2, silhouette = 0.372) in CLR space. The first cluster comprises individuals with the IDs Cd6/M450, Cd7/M450, Cd1/M550, and Cd2/M550, and the second cluster comprises individuals Cd2/M450, Cd3/M450, Cd4/M450, Cd5/M450, Cd8/M450, and Cd9/M450. The greatest contribution to the differentiation of oilfish individuals by the lipid profile along the first principal axis (PC1) is made by minor PL classes (PI, LPC, and SM) together with multifunctional CholEs and waxes. On the other hand, differentiation along the second principal axis (PC2) is primarily generated by DAGs, CholEs, MAGs, PE, and PS.

Statistical modeling of the whole-body lipidome distribution in little Baikal oilfish explained 81.8% of the total variance (PC1—58.8%, PC2—23.0%) and, similarly to muscle tissue, returned a two-cluster arrangement (Ward’s method: k = 2; silhouette = 0.524), in which ten specimens are clustered together, while one specimen (Cd1/WB550) featured a distinct lipid profile (Figure 3). The distribution of the clusters along PC1 is generated by PI, PS, PC, TAGs, and MAGs, and that along PC2 is generated by MAGs, CholEs, DAGs, waxes, and NEFAs. It is thus demonstrated that oilfish individuals exhibit reproducible clustering based on the lipid profiles of both muscles and whole body. The differentiation between the profiles appears to be associated with coordinated variation between membrane PLs (PI, PS, PC, LPC, SM) and neutral lipid fractions (TAGs, MAGs, CholEs, waxes), indicating inter-correlation between changes in structural and energetic lipids in *C. dybowskii* in response to environmental impacts.

Statistical analysis for correspondence between individual lipid profiles and the size and weight parameters of oilfish and their sampling depth based on the principal components plane (PC1 and PC2), with the output tested for significance via PERMANOVA, established the absence of significant correlations between the lipidome of *C. dybowskii* and the said non-lipid variables. The body length and weight had no significant correlations with the lipid profile of either muscle tissue (R^2^ ≤ 0.034; *p*-value = 0.729) or whole body (R^2^ ≤ 0.135; *p*-value = 0.274). The depth, as an integrated factor comprising the hydrostatic pressure and temperature impacts, did have some effect on the lipid profile of muscles, as evidenced by a moderate, statistically unsupported trend (R^2^ = 0.238; *p*-value = 0.127). No such relationship was found for individuals subjected to whole-body lipid analysis (R^2^ = 0.112; *p*-value = 0.337). PERMANOVA also revealed no significant effect of depth on the lipid profile of *C. dybowskii* (muscles: F = 2.134, *p*-value = 0.119; whole body: F = 0.984, *p*-value = 0.400), which is in agreement with non-parametric tests for correlations and differences between clusters. Altogether, the outcome of the multivariate statistical tests suggests that depth-related environmental factors influence the muscle tissue lipidome in little Baikal oilfish. Yet, the current sample size appears insufficient for the comprehensive identification of specific environmental controls and the scope of their effect on the whole-body lipidome in this species.

**Figure 4:**
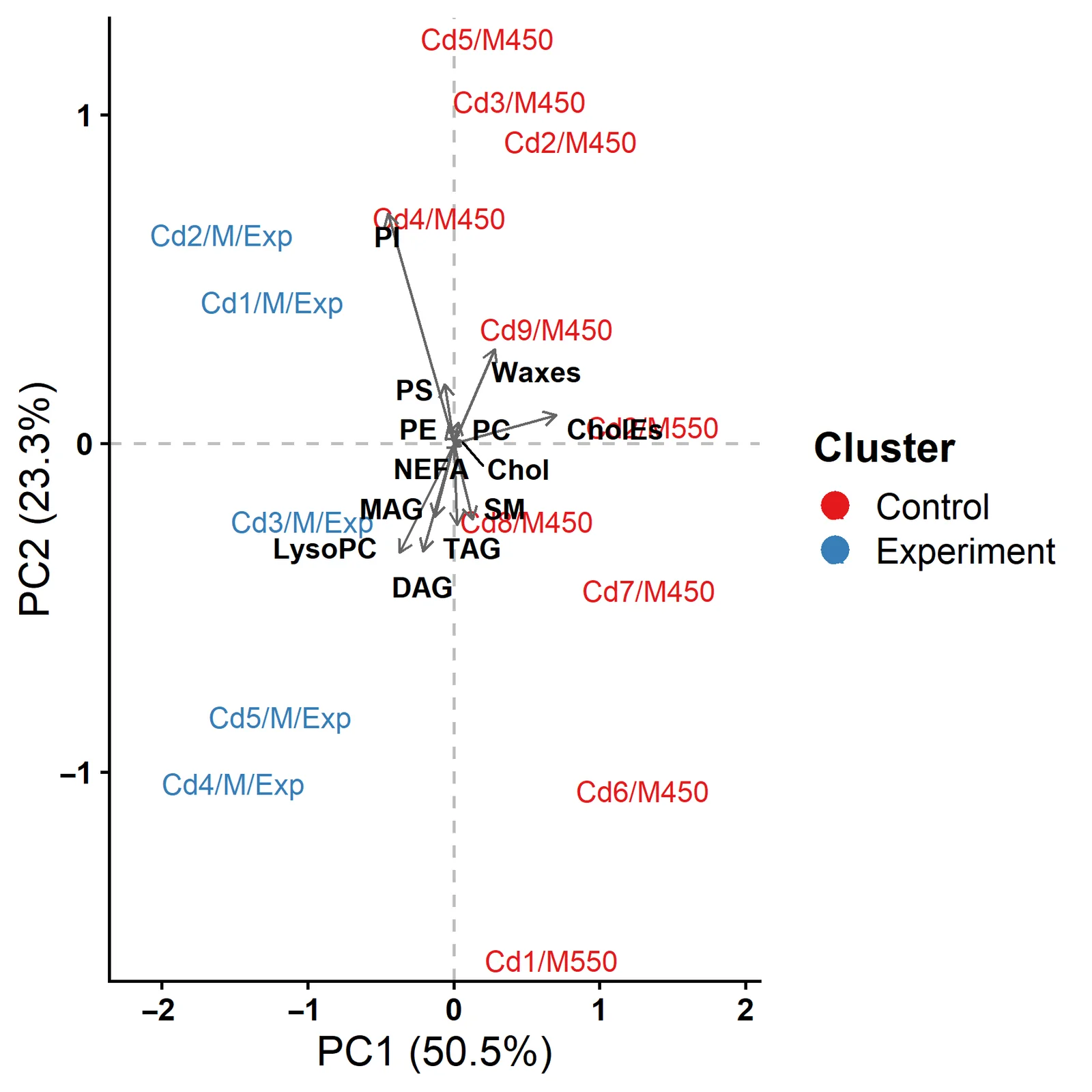
PCA ordination of *C. dybowskii* individuals of the control and experimental groups based on the muscle tissue lipid profile in CLR space, with clustering via Ward’s method. Note: TLs—total lipids, PLs—total phospholipids, TAGs—triacylglycerols, CholEs—cholesterol esters, waxes—wax esters, Chol—cholesterol, MAGs—monoacylglycerols, DAGs—diacylglycerols, NEFAs—non-esterified (free) fatty acids, PC—phosphatidylcholine, PE—phosphatidylethanolamine, PI—phosphatidylinositol, PS—phosphatidylserine, LysoPC—lysophosphatidylcholine, SM-sphingomyelin. Fish identity legend for the experiment (Cd2/M/Exp as the example): Cd—*C. dybowskii*, 2—ID number of fish, M—muscles, Exp—experiment.

At the same time, the experimental transfer of several oilfish individuals from the 450–550 m depth horizon into a controlled-environment tank (3–6 °C temperature, 5‰ salinity) for 24 h was associated with an increase in the content of metabolism-associated forms of acylglycerols (MAGs, DAGs, TAGs), as well as membrane PE, Chol, and LysoPC in their muscle tissue (Table 2). These differences are confirmed via PERMANOVA—F = 9.797 with *p*-value = 0.001. The principal component analysis of the distribution of oilfish individuals in CLR space (Figure 4) demonstrates a steady differentiation of the analytical specimens of muscle tissue into two clusters (Ward’s method: k = 2, silhouette = 0.424), which explains 73.8% of the total variance (PC1 = 50.5%, PC2 = 23.3%). The first axis (PC1) is shaped by CholEs, PI, LysoPC, waxes, and DAGs, while PC2 is primarily formed by PI, LysoPC, DAGs, waxes, and TAGs. The explained share of data variation on the PC1–PC2 plane remains high (R^2^ = 0.579, *p*-value (permutation) = 0.001), and individuals from the experimental group form a steady vector for differentiation of the sample into clusters.

**Figure 5:**
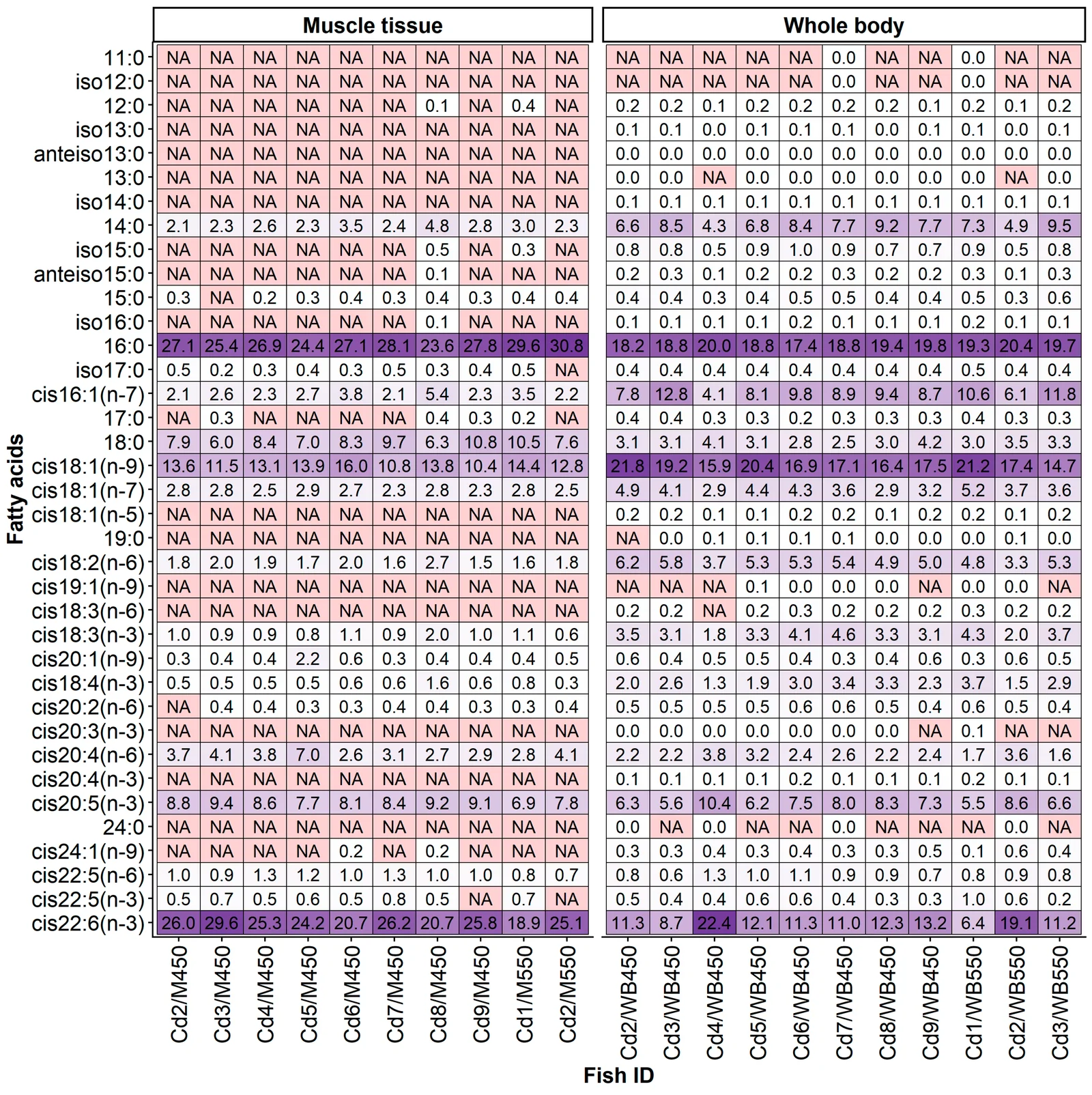
Heatmap of the muscle-tissue and whole-body lipid profiles of *C. dybowskii* from Lake Baikal. Note: fish identity legend (Cd2/M450 as the example): Cd—*C. dybowskii*, 2—ID number of fish, M—muscles (WB—whole body), 450 (or 550)—capture depth (m). The purple color gradient reflects the quantity of the fatty acid in the tissue/body. “NA” (not available) in red cells indicates the FA is absent from the spectrum.

**Figure 6:**
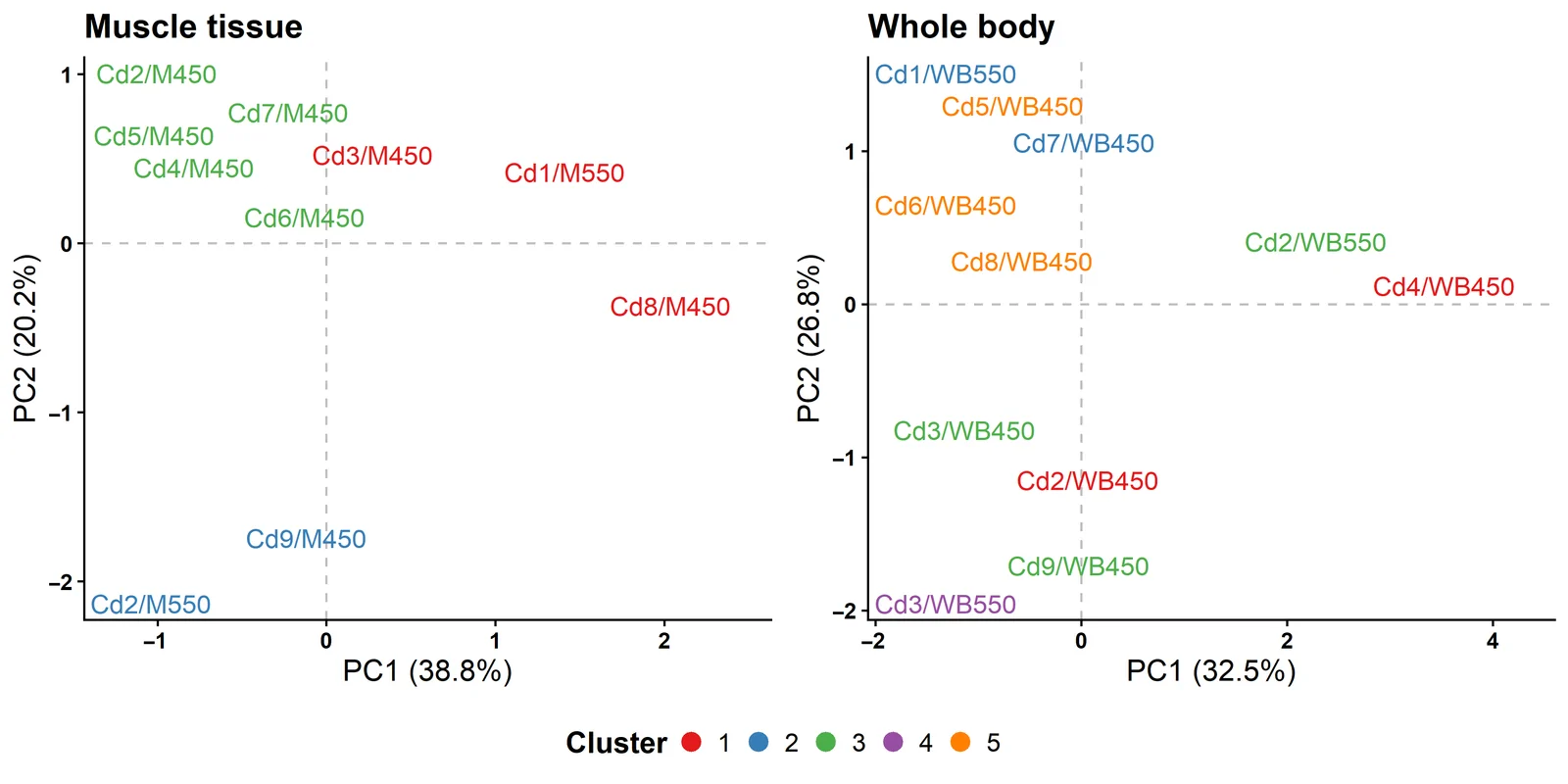
PCA ordination of *C. dybowskii* individuals based on the muscle-tissue and whole-body FA profiles in CLR space, with clustering via Ward’s method. Note: fish identity legend (Cd2/M450 as the example): Cd—*C. dybowskii*, 2—ID number of fish, M—muscles (WB—whole body), 450 (or 550)—capture depth (m).

Having separated individual FAs from TLs, we identified a total of 24 FAs in muscle tissue and 37 FAs in whole-body samples (Figure 5). Multivariate analysis of the FA profile of *C. dybowskii* muscles via PCA with Ward’s clustering collectively explained 59.0% of the total variance (PC1 = 38.8%, PC2 = 20.2%), with individuals forming three clusters (k = 3, silhouette = 0.483): cluster 1—Cd3/M450, Cd8/M450, Cd1/M550; cluster 2—Cd9/M450, Cd2/M550; cluster 3—Cd2/M450, Cd4/M450, Cd5/M450, Cd6/M450, Cd7/M450 (Figure 6). At the same time, whole-body lipidome analysis returned five distinct clusters of individuals (k = 5, silhouette = 0.415), while the explained variance share was similar—59.3% (PC1 = 32.5%, PC2 = 26.8%): cluster 1—Cd2/WB450, Cd4/WB450; cluster 2—Cd7/WB450, Cd1/WB550; cluster 3—Cd3/WB450, Cd9/WB450, Cd2/WB550; cluster 4—Cd3/WB550; cluster 5—Cd5/WB450, Cd6/WB450, Cd8/WB450 (Figure 6). These models were found to differ markedly in the contributions of variables to the principal component axes: in the ‘muscle’ model, the greatest contributions to PC1 are made by 17:0, iso15:0, 12:0, anteiso15:0, and iso16:0, and the greatest contributions to PC2 are made by cis22:5(n-3), 17:0, iso17:0, cis24:1(n-9), and cis20:2(n-6), while in the ‘whole-body’ model, PC1 is primarily shaped by 24:0, 13:0, cis19:1(n-9), cis18:3(n-6), and cis20:3(n-3), and PC2 is primarily shaped by cis19:1(n-9), 24:0, 11:0, iso12:0, and cis20:3(n-3). No statistically supported associations, either qualitative or quantitative, were detected for the impact of size and weight parameters and capture depth on the FA profiles of muscle tissue and whole-body samples of *C. dybowskii*. On the other hand, a close analysis of the mean carbon chain length and degree of unsaturation in the oilfish samples revealed the deposition of longer-chain and more highly unsaturated FAs (ACL = 19, UI = 1.98–2.76) in muscle tissue versus whole body (ACL = 18, UI = 1.64–2.53).

The assessment of alignment between multivariate distributions of individuals by lipid classes and by the FA profile did not reveal any common structure of distances between individuals or cluster partitioning coincidence between the profiles. The comparison of the configurations based on the two principal components (PC1 and PC2) via Procrustes analysis showed low geometric similarity of the lipid and FA spaces (muscles: similarity = 0.21; whole body: similarity = 0.18). The correlation analysis of distance matrices (Spearman’s method) also found no alignment between the lipid and the FA profiles of *C. dybowskii* (muscles: r = −0.092, *p*-value (perm) = 0.692; whole body: r = −0.037, *p*-value (perm) = 0.522). Clustering was not aligned between the two profiles either—ARI values for k = 2 were −0.106 and 0.038, respectively; AMI values when comparing the lipid profile’s k = 2 with the FA profile’s optimal k were −0.188 and 0.019. Thus, there was clear differentiation in the structure of oilfish grouping by the lipid and the FA profiles.

**Figure 7:**
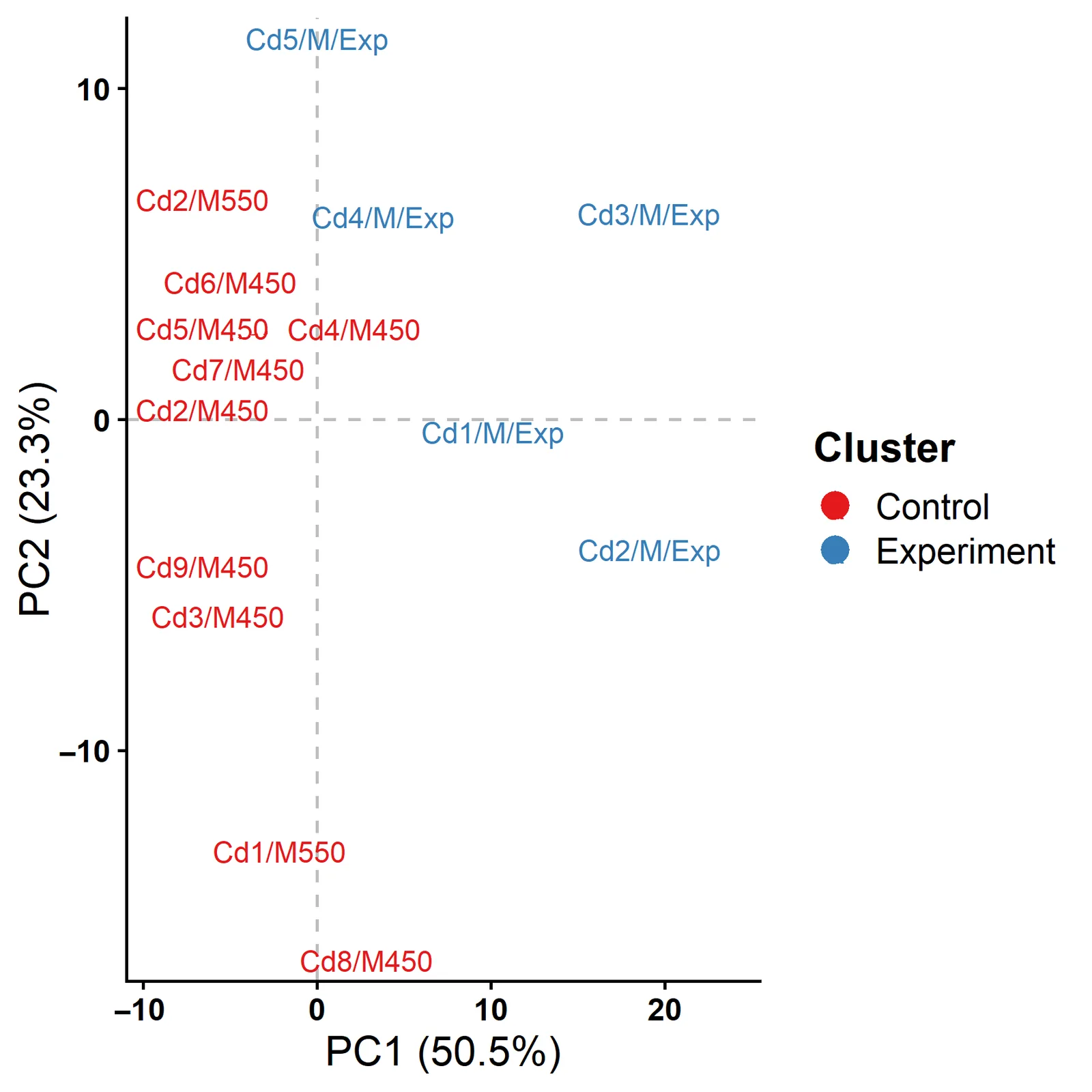
PCA ordination of *C. dybowskii* individuals of the control and experimental groups based on the muscle tissue FA profile in CLR space, with clustering via Ward’s method. Note: fish identity legend for the experiment (Cd2/M/Exp as the example): Cd—*C. dybowskii*, 2—ID number of fish, M—muscles, Exp—experiment.

The experimental transfer of oilfish to a controlled environment for 24 h also had a notable effect on the FA profile of muscle tissue. Multivariate PCA preceded by CLR transform revealed an explicit structuring of the ‘control’ and ‘experiment’ individuals along the first principal axis (PC1 = 33.9%), emerging due to oppositely directed trends in the contributions of long-chain unsaturated FAs, chiefly cis20:5(n-3) and cis22:6(n-3), as well as saturated fatty acids (SFAs) and monounsaturated fatty acids (MUFAs) (Figure 7). The second principal axis (PC2 = 16.5%) represents the distribution by the quantities of methyl-branched FAs and odd-chain components in muscles. Although fish of the control and the experimental groups demonstrate some differentiation with partial overlap in the PC1–PC2 space, multivariate analysis of similarities (ANOSIM) showed very strong differentiation based on the muscle FA profile (R = 1, *p*-value = 0.004). Clustering of the mixed control/experiment sample using Ward’s method returned three steady clusters, which reflect differences in the dominant metabolic pathways in oilfish muscles. It is noteworthy that individuals of the two groups were unevenly distributed between clusters, i.e., oilfish of the control group more often belonged to the cluster with a higher content of long-chain polyunsaturated fatty acids (PUFAs), whereas the shift in fish of the experimental group was more towards MUFAs and branched-chain/odd-chain FAs. PERMANOVA corroborated significant differences (*p*-value < 0.05) between the groups of fish in terms of the muscle FA profile. Importantly, the statistical effect of between-group differences was significant, while the explained share of variance was moderate, which is typical of experiments in ecological biochemistry and points to systemic alteration of the metabolic (FA) composition of muscle tissue.

The combined analysis of the principal component loadings, clustering structure, and vectors of distribution shift for oilfish of the experimental group has led to the identification of several functional FA groups, which can be regarded as biomarkers of the mechanisms of adaptation to environmental changes within the experiment. Specifically, a notable decline was observed in the cis20:5(n-3) (from 8.49 to 7.78% of total FAs) and cis22:6(n-3) (from 25.22 to 9.68%) PUFA content in *C. dybowskii* muscles upon transfer from deep water to a controlled environment (tank). The decline in long-chain n-3 PUFAs is evidenced both by a rise in the content of the intermediate cis22:5(n-3) FA (from 0.54 to 0.96%) and an increase in cis16:1(n-7) (by +235%) and cis18:1(n-9) (by +30%) in muscles.

Additionally, we observed a considerable rise in odd-chain FAs (17:0) and branched-chain FAs (iso15:0, iso17:0, anteiso15:0) in oilfish muscles upon transfer to a controlled environment (+60–180% versus the control). At the same time, the amount of the 15:0 FA remained unchanged, with a minor reduction in its share among all FAs (from 2.61 to 2.58%).

## 4. Discussion

The lipid profile in fishes of the genus *Comephorus* is characterized by pronounced variability in both TL accumulation and in the ratios of individual lipid classes, as demonstrated in a number of previous studies [20,25,26]. The known average TL content in *C. dybowskii* muscle tissue is 2.6% dry weight [20], which is in agreement with our results. The TL accumulation determined in our study is at a low to moderate level, characteristic of pelagic fish species [20]. On the other hand, the wide range of variation in the contents of both TLs and specific lipid classes among fish individuals indicates the high inter-individual variability of the biochemical status at the structural and energy-metabolism level. Such variability is regarded as typical for pelagic and deep-water fishes and can reflect differences in the physiological status, stage of the reproductive cycle, intensity of vertical migrations, habitat depth, and/or relatively recent diet [27,28].

Analysis based on a combination of multivariate statistical methods identified consistent patterns in the variable lipid profile of the muscle tissue and whole body of the fish. Statistical offsetting of the contribution of random and individual-specific variations in certain lipid classes (including correlated ones) enabled the structuring and clustering of lipidome variability among oilfish individuals, exposing the dominant mechanisms for the organism’s lipid metabolism organization. At the same time, the lack of significant correlations between the muscle and whole-body lipid profiles and the fish size and weight uncouples the sample from age-related changes and individual morphometric parameters. Ultimately, the detected two-cluster structure of the oilfish lipidome reflects at least two persistent variants of the lipid-metabolism dimension of the physiological and biochemical mechanisms of adaptation to the ambient conditions.

One such mechanism is the compensatory remodeling of cell membranes in response to depth-related environmental controls through interactions of SM/Chol-enriched domains (lipid rafts) and minority PLs (PI, LysoPC) in myocytes. Hydrostatic pressure and temperature co-vary with depth, and in the present study, these two factors are considered unified, and depth is interpreted as an integrated environmental factor. Intermolecular interactions between the Chol steroid nucleus and SM are known to initiate stable liquid-ordered domains, making membranes locally more robust to various mechanical impacts, including lateral compression under elevated hydrostatic pressure [29,30,31]. Adaptation of membrane lipids to combinations of low temperature and elevated hydrostatic pressure has recently been recognized as a common feature of deep-water organisms, further emphasizing the importance of the lipid composition in maintaining membrane integrity under extreme environmental conditions [32]. The identified SM variations, correlating with metabolism-associated esterified cholesterol (CholEs) in oilfish muscles, may indicate the formation of such rafts in myocytes to locally regulate bilayer order without altering the overall membrane architecture. On the other hand, it has been established that the rigidity of such domains is directly controlled by recruiting PI and LysoPC molecules along the edges of the rafts [31,33,34]. As demonstrated experimentally, PI-enriched areas in the lipid bilayer serve as “flexible regions” which, together with LysoPC molecules, buffer the local rigidity of liquid-ordered domains (lipid rafts) [31,33]. Thus, this combination of raft (SM/Chol) and non-raft (LysoPC, PI) microdomains is consistent with a potential adaptive mechanism that may contribute to maintaining membrane integrity and the functionality of membrane-bound proteins under depth-related environmental impacts [3,30,35].

As opposed to the first principal component (PC1), which primarily represents membrane characteristics related to structural adaptations, the second principal component (PC2), composed of DAG, MAG, and CholE molecules, as well as PE and PS, pointed to another mechanisms of lipid profile organization, related to the dynamics of metabolic processes. Analysis showed that the key contributors to PC2 in muscle tissue are DAGs and MAGs—intermediate products in lipid metabolism. It is known that these molecules can perform a dual function of both metabolic intermediates and signaling molecules, regulating a wide spectrum of cellular processes, including the activity of lipases (such as the hormone-sensitive or adipocyte TAG lipase [36]), membrane remodeling, and cell response to stress [11]. Their variability reflects the balance between neutral lipid synthesis and breakdown, as well as the renewal rate of the PL structure of cell membranes.

At the same time, PE and PS, which also varied along the PC2 axis, are associated with membrane remodeling and facilitating lipid bilayer plasticity. Due to the pronounced geometric asymmetry between their head groups and acyl radicals, these PLs are distributed to the inner leaflet of the lipid bilayer [37]. Owing to this ‘conical’ geometry, they can form a leaflet with negative curvature [37]. These PLs are mainly localized in dynamic areas of the membrane, avoiding the rigid packing of SM/Chol rafts [34,38], potentially facilitating functional adjustment of the lipid bilayer and helping to offset local mechanical stresses.

The variations in CholEs in oilfish muscles along the PC2 axis, in combination with variations in MAGs and DAGs, suggest that these processes are closely correlated, with CholE mobilization triggering the simultaneous release of both the structural component of membranes (non-esterified Chol) and energetic FAs. It is known that Chol is deposited in the form of CholEs, which play a key role in regulating its intracellular pool, securing a balance between membrane Chol and its storage [39].

Considering the entirety of the variations detected in individual lipid classes, we can discuss a two-tier system of adaptations in *C. dybowskii* muscles, where the structural stability of membrane domains is synchronized with dynamic regulation of the lipid metabolism and membrane remodeling. Such organization is a solution for membrane resilience to depth-related environmental impacts while, at the same time, maintaining the functional plasticity of muscle tissue, which is a vital prerequisite for the viability of a deep-water pelagic species performing vertical migrations.

As opposed to the muscle tissue of little Baikal oilfish, where the lipid profile variability is largely governed by membrane remodeling, the local adaptation of cells, and the deposition of essential lipidic compounds, their whole-body lipidome reflects the combined contribution of a variety of tissues. The allocation of lipids among tissues in fish is known to be heterogeneous and directly dependent on their functional specializations [40]. Nevertheless, we found common structural features in the lipid composition variability in whole-body and muscle-tissue samples of *C. dybowskii* in what concerns alignment between membrane organization and energy metabolism. Specifically, they retained the lipidome component associated with intermediate products of lipid metabolism (DAGs, MAGs) and storage lipids (ChoEs and TAGs). This commonality highlights coordination between lipid-metabolism processes and their role in regulating the organism’s physiological status in response to environmental changes [11]. To specify, the contribution of the energetic TAGs is higher in the whole body than in muscles, suggesting that TAGs are an important energy depot for oilfish at the whole-organism level, whereas the dominance of low-density CholEs and waxes in muscle tissue is consistent with a potential role in buoyancy regulation [8,40].

The experimental transfer of *C. dybowskii* individuals from the natural habitats of Lake Baikal into controlled-environment tanks revealed patterns of muscle lipidome transformation that may reflect adaptive metabolic responses. The most noticeable changes occurred in acylglycerols (MAGs, DAGs, TAGs) and in PE, LysoPC, and Chol. In particular, the observed rise in the minority MAGs and DAGs points to the substantial activation of lipid metabolism and the acceleration of lipolysis after the transfer. These compounds are recognized as universal intermediate metabolites, which reflect the lipid metabolism rate and lipase system activity [11], and alterations of their amounts in tissues and organs are characteristic of the stress-induced metabolic transformation in fish in response to changes in temperature and other ambient conditions. In combination with elevated TAGs, these parameters indicate a transition to a lipid metabolism state involving the simultaneous activation of both lipogenesis and lipolysis. This ‘cyclic’ pattern of lipid metabolism functioning is considered to be typical under physiological stress [41].

In parallel, remodeling of the cellular lipid bilayer also occurred, as evidenced by a rise in LysoPC, which indicates activation of the phospholipase pathway and the intensified renewal of membrane structures [42,43]. In particular, a rise in PE has been reported to increase the myocytic membrane curvature, which is associated with higher plasticity of membranes, as well as their fusion and remodeling [44]. On the other hand, the lipid bilayer is stabilized under water temperature and salinity change through the recruitment of Chol molecules, which regulate the order and rigidity of membranes, thus influencing their permeability and resistance to external impacts [39].

Changes in the composition of lipid classes alter not only their ratios but also the FA composition of structural and storage lipids. Since FAs determine the key physicochemical properties of lipids, thereby regulating membrane fluidity, energy capacity, and resistance to externalities, the analysis of their profile has provided new insights into the mechanisms of adaptation to environmental impacts. Namely, the differentiation of oilfish individuals into disjoint clusters differing in the content of both short- and medium-chain SFAs and long-chain PUFAs is a direct indication that alternative strategies of lipid metabolism organization exist at the FA-profile level.

We found that a key feature of the FA composition of oilfish muscles was the lower content of long-chain and unsaturated FAs compared to their whole-body content. This feature reflects the functional specialization of muscle tissue, in which the molecular forms of lipids secure the stability and functionality of biological membranes in deep-water habitats. It is known that long-chain PUFAs, especially of the n-3 and n-6 series, play a major role in maintaining cell membrane rigidity, reducing the packing density of the lipid bilayer under high pressure and low temperatures [4,45]. The functional importance of n-3 and n-6 PUFAs for biological membrane organization has been repeatedly emphasized, as these molecules influence membrane properties through their distinct effects on lipid packing and bilayer dynamics [46,47]. Notably, the highest variability was exhibited by the cis22:5(n-3) and cis20:2(n-6) FAs, suggesting they may be actively involved in compensatory adaptation mechanisms for maintaining adequate cell membrane fluidity. The observed distribution of these components in muscle tissue is consistent with predictions of the homeoviscous adaptation mechanism, according to which aquatic organisms regulate membrane fluidity by modifying lipid unsaturation degrees [48,49]. As is known, the effect of pressure and temperature in most cases have a similar effect on the biomembrane of cells; however, there is also evidence of the partially independent influence of these abiotic factors on the FA composition, supporting the concept that lipid metabolism may respond differently to multiple physical factors characteristic of deep-water environments [50]. From a physiological perspective, the accumulation of long-chain PUFAs may contribute to membrane functionality under low-temperature and high-pressure conditions. Due to their molecular structures, these FAs reduce the lipid packing density and increase bilayer flexibility, thereby supporting membrane-protein activity, transmembrane transport processes, and enzymatic reactions associated with cellular metabolism. Consequently, the observed FA composition is consistent with biochemical adjustments that help preserve membrane performance in the deep-water environment inhabited by *C. dybowskii*. On the other hand, the lack of alignment between the lipid and the FA profiles in muscles of little Baikal oilfish suggests that the FA profile does not directly follow the distribution of lipid classes (as described previously), but is shaped by partially autonomous mechanisms leveraging intracellular homeostasis. Similar conclusions have previously been drawn regarding other aquatic organisms (e.g., [51]), demonstrating that the FA composition is orchestrated not only by the lipid profile but also by specific metabolic and physiological processes.

We also found that the greatest contribution to the FA-profile variation among fish individuals comes from SFAs, including odd-chain compounds (e.g., 17:0), as well as branched-chain forms (iso- and anteiso-FAs). The presence of these components in tissue represents specific features of the lipid metabolism in the body of fish and indicates the microbial input to oilfish FA profiling, since such FAs are traditionally regarded as biomarkers of bacteria consumed with food or acquired through contact with the microbiota [52]. The variability of these components in muscles may reflect variations in the trophic status of *C. dybowskii* and in the rate of metabolic processes or degree of exposure to the microbial community.

The whole-body FA composition in *C. dybowskii* exhibits a more complex and multi-tiered structure of variability than that of muscle tissue, reflecting the contributions of different organs and tissues to the organism’s FA profile. Multivariate analysis returned five steady clusters of fish, differentiated by SFAs, as well as PUFAs. The contributions of individual FAs to the principal components (PC1 and PC2) in this case differed notably from the pattern in muscle tissue, suggesting that FA-regulation mechanisms at the systemic level are different.

As opposed to muscle tissue, where the structural role of lipid components dominates, the whole-body FA profile represents the integrated effect of the metabolic, energy, and structural functions of this molecule class, as corroborated by a shorter average hydrocarbon chain length and a lower degree of unsaturation compared to muscle tissue. The variations detected in SFAs, MUFAs, and PUFAs along the first principal component (PC1) apparently serve for balancing the storage and structural functions of FAs at the organismal level. It is known that SFAs are capable of densely packing PLs into membranes and promoting lipid bilayer stability and are used in energy and storage metabolism, whereas MUFAs perform a transitional function, ensuring that membranes are relatively fluid while retaining stability, and are actively used in energy metabolism [3]. At the same time, variations along the PC2 axis are similar to variations in muscle tissue long-chain SFAs, including odd-chain compounds (e.g., 17:0) and branched-chain forms (iso- and anteiso-FAs). This similarity corroborates the findings regarding FA metabolism in the fish body, as well as the microbial contribution to FA profiling, which appears to be of an exogenous (trophic) nature. Therefore, variation in the whole-body fatty acid composition likely reflects the organism-level regulation of energy allocation, where fatty acids simultaneously participate in membrane maintenance and in the formation of metabolically available energy reserves.

The experiment with the transfer of *C. dybowskii* from their natural habitats in Lake Baikal to controlled-environment tanks entailed a pronounced transformation of the FA profile of fish muscles, reflecting the organism’s transition from a steady adapted state to stress-induced metabolic rearrangement. The marked reduction in the share of long-chain PUFAs (cis20:5(n-3) and cis22:6(n-3)) with a simultaneous rise in MUFAs and intermediate FAs indicates that a set of interrelated adaptation mechanisms has been activated to rapidly remodel membranes to fit the new ambient conditions. Apparently, one of these mechanisms is the elevation of the degree of unsaturation of FAs in muscle cell membranes under changes in temperature and pressure to enhance the lipid bilayer’s stability and support its function [3,48]. Research has demonstrated that temperature stress can involve a reduction in PUFAs with a simultaneous rise SFAs in an organism [53,54]. It is worth noting, in this context, that *C. dybowskii* from Lake Baikal also exhibited variations precisely in SFAs both in muscle tissue and at the whole-body level, suggesting that this mechanism may often be used to respond to changes in the environment, in particular during vertical migrations. Hence, this mechanism may represent a mechanism facilitating the rapid adjustment of membrane properties to adjust to the modified physicochemical conditions. Comparable shifts in the FA composition have been observed in fish exposed to thermal stress, where relatively constant FA profiles and the remodeling of lipid metabolic pathways are realized as a part of adaptive physiological plasticity [54].

A simultaneous decline in long-chain n-3 PUFAs (in particular cis20:5(n-3) and cis22:6(n-3)) in muscles upon transfer to a controlled environment may be associated with their high sensitivity to peroxidation, since these components are the main lipid peroxidation substrates, and their excess under stress may result in damage to cellular structures [55]. A reduction in the share of these molecules in membrane lipids can be viewed as a protective mechanism designed to mitigate oxidative damage. On the other hand, a rise in the share of MUFAs (cis16:1(n-7), cis18:1(n-9)) in oilfish muscles upon the change in the environmental conditions points to activation of the Δ9-desaturase pathway. It is known that MUFAs are similar to PUFAs in that they can lower the lipid melting temperature (by reducing the Van der Waals forces of interactions between neighboring FAs), providing the required membrane fluidity, but this group of molecules is less oxidizable than PUFAs [56]. Hence, a rise in the share of MUFAs can be viewed as a more energy-efficient and reliable method of regulating membrane properties under stress. Such a shift may simultaneously support appropriate membrane fluidity and reduce the susceptibility of membrane lipids to oxidative damage, providing a potentially advantageous balance between membrane functionality and oxidative stability during environmental stress.

The rise in the content of odd-chain FAs (17:0) and branched-chain FAs (iso15:0, iso17:0, anteiso15:0) in oilfish muscles upon transfer from the lake to controlled-environment tanks is specifically worth mentioning. As reported elsewhere, these compounds are classical bacterial biomarkers [52], and their augmentation may evidence microbial input to the organism’s stress-induced response. Furthermore, the removal of oilfish from deep water and their abrupt placement into a controlled environment could provoke a special sort of stress, which, in the absence of feeding, can trigger a slowdown of intestinal peristalsis, causing increased contact of the epithelium with the microbiota and, ultimately, the partial lysis of bacterial cells [57]. However, fish of the experimental group feeding on detritus dropping accidentally from the trawl during the transfer can be ruled out since there were no significant changes in the 15:0 FA content and because the renewal of the FA profile of the muscle tissue was relatively slow.

## 5. Conclusions

This study suggests that the lipid and FA profiles of the little Baikal oilfish (*C. dybowskii*) represent a multi-tiered system of biochemical responses associated with adaptation to depth-related environmental conditions in Lake Baikal. Individual lipid classes may contribute to the structural and metabolic organization of cells, including SM/Chol rafting, the regulation of membrane plasticity through minority PLs, and the lipid-metabolism dynamics expressed in acylglycerol and CholE variations. These mechanisms potentially serve to maintain a balanced membrane organization and lipid metabolism. The FA profile appears to perform a complementary metabolism-associated level of biochemical adaptation characterized by variations in the acyl chain length and degree of unsaturation. The accumulation of long-chain PUFAs is consistent with mechanisms that maintain membrane functionality under low temperatures and elevated hydrostatic pressure. The observed stress-induced response was associated with variation in SFA and MUFA contents. Furthermore, variations in saturated, odd-chain, and branched-chain FAs indicate the organism’s metabolic characteristics and the potential microbial contribution. The lack of alignment between the lipid and the FA profiles suggests a degree of functional independence between these regulatory levels.

Thus, the results support the hypothesis that adaptation in *C. dybowskii* involves the hierarchical organization of biochemical responses according to these membrane-associated lipids, lipid-metabolism dynamics, and FA compositions, which vary in a coordinated and tightly associated manner under changing environmental conditions. These findings broaden our understanding of the mechanisms of biochemical adaptation in deep-water organisms and highlight the key role of the lipid metabolism in regulating their physiological state.

## Figures and Tables

**Table 1:** Qualitative and quantitative characteristics of the lipid profile (% dry weight) of muscle tissue and whole-body samples of little Baikal oilfish *Comephorus dybowskii*.

Fish ID	TLs	PLs	TAGs	CholEs	Waxes	Chol	MAGs	DAGs	NEFAs
Muscle tissue									
Cd2/M450	3.08	0.46	0.58	0.80	0.46	0.39	0.02	0.07	0.29
Cd3/M450	3.07	0.42	0.70	0.71	0.51	0.38	0.02	0.06	0.28
Cd4/M450	4.82	0.64	0.83	0.89	1.20	0.63	0.04	0.12	0.47
Cd5/M450	4.46	0.60	0.75	0.58	1.44	0.57	0.03	0.12	0.36
Cd6/M450	0.77	0.11	0.19	0.17	0.10	0.10	0.01	0.02	0.07
Cd7/M450	7.32	1.23	1.33	2.03	0.77	0.90	0.02	0.17	0.87
Cd8/M450	3.32	0.36	1.02	0.58	0.53	0.39	0.02	0.13	0.28
Cd9/M450	1.39	0.22	0.24	0.34	0.21	0.18	0.01	0.03	0.17
Cd1/M550	1.46	0.18	0.34	0.34	0.19	0.19	0.02	0.06	0.14
Cd2/M550	3.00	0.30	0.48	1.30	0.36	0.29	0.02	0.02	0.22
Whole body									
Cd2/WB450	2.28	0.30	1.17	0.06	0.33	0.24	0.01	0.06	0.11
Cd3/WB450	10.61	1.12	3.73	0.50	2.51	1.37	0.03	0.42	0.92
Cd4/WB450	3.29	0.34	1.58	0.10	0.53	0.37	0.02	0.13	0.24
Cd5/WB450	8.38	0.72	4.42	0.19	1.29	0.90	0.04	0.35	0.49
Cd6/WB450	14.75	1.25	7.67	0.43	2.03	1.66	0.09	0.74	0.88
Cd7/WB450	11.65	1.03	6.07	0.41	1.61	1.29	0.05	0.51	0.68
Cd8/WB450	11.49	1.03	5.80	0.37	1.94	1.17	0.05	0.43	0.71
Cd9/WB450	10.36	0.95	4.84	0.52	1.82	1.14	0.02	0.21	0.85
Cd1/WB550	11.42	0.92	7.06	0.21	1.32	1.01	0.03	0.23	0.64
Cd2/WB550	3.66	0.39	1.39	0.16	0.89	0.44	0.01	0.11	0.27
Cd3/WB550	10.76	1.13	5.37	0.48	1.81	1.06	0.04	0.29	0.59

Note: TLs—total lipids, PLs—total phospholipids, TAGs—triacylglycerols, CholEs—cholesterol esters, waxes—wax esters, Chol—cholesterol, MAGs—monoacylglycerols, DAGs—diacylglycerols, NEFAs—non-esterified (free) fatty acids. Fish identity legend (Cd2/M450 as the example): Cd—*C. dybowskii*, 2—ID number of fish, M—muscles (WB—whole body), 450 (or 550)—capture depth (m).

**Table 2:** Changes in the contents of individual lipid classes in *C. dybowskii* muscles after transfer to a controlled environment.

Lipid	*C. dybowskii* (Control)	*C. dybowskii* (Experiment)	Δ Median	*p*-Value	FDR	r
MAG	0.02	0.06	+0.04	6.66 × 10^−4^	0.0087	+1.00
DAG	0.07	0.25	+0.18	0.00133	0.0087	+0.96
LysoPC	0.002	0.012	+0.010	0.00266	0.0115	+0.92
PE	0.12	0.28	+0.16	0.00799	0.0260	+0.84
TAG	0.64	1.37	+0.73	0.01265	0.0314	+0.80
Chol	0.38	0.75	+0.37	0.01931	0.0314	+0.76

Note: the table includes only those lipid classes for which differences between groups are statistically supported. “*C. dybowskii* (Control)”—median lipid content in individuals sampled from 450—550 m depths; “*C. dybowskii* (Experiment)”—median lipid content in individuals transferred for 24 h from 450—550 m depths to controlled-environment tanks; Δ median—difference between medians; *p*-value—significance of inter-group differences determined via Wilcoxon—Mann—Whitney test; FDR—correction for multiple simultaneous comparisons (Benjamini—Hochberg, BH-correction—values ≤ 0.05 mean the result remains significant after correction); r—metric of effect size (related to the U test, takes values between −1 and +1). TAGs—triacylglycerols, Chol—cholesterol, MAGs—monoacylglycerols, DAGs—diacylglycerols, PE—phosphatidylethanolamine, LysoPC—lysophosphatidylcholine.

## Data Availability

All data are presented in the paper.
